# Performance Optimization of Wearable Printed Human Body Temperature Sensor Based on Silver Interdigitated Electrode and Carbon-Sensing Film

**DOI:** 10.3390/s23041869

**Published:** 2023-02-07

**Authors:** Aisha M. Al-Qahtani, Shawkat Ali, Arshad Khan, Amine Bermak

**Affiliations:** 1 Division of Information and Computing Technology, College of Science and Engineering, Hamad Bin Khalifa University, Qatar Foundation, Doha 5825, Qatar; 2 Sensors Lab, Advanced Membranes and Porous Materials Center, Computer, Electrical and Mathematical Science and Engineering Division, King Abdullah University of Science and Technology (KAUST), Thuwal 23955-6900, Saudi Arabia

**Keywords:** human body temperature sensor, flexible biosensor, printed IDE, silver nanoparticles, inkjet material printer, carbon black

## Abstract

The human body’s temperature is one of the most important vital markers due to its ability to detect various diseases early. Accurate measurement of this parameter has received considerable interest in the healthcare sector. We present a novel study on the optimization of a temperature sensor based on silver interdigitated electrodes (IDEs) and carbon-sensing film. The sensor was developed on a flexible Kapton thin film first by inkjet printing the silver IDEs, followed by screen printing a sensing film made of carbon black. The IDE finger spacing and width of the carbon film were both optimized, which considerably improved the sensor’s sensitivity throughout a wide temperature range that fully covers the temperature of human skin. The optimized sensor demonstrated an acceptable temperature coefficient of resistance (TCR) of 3.93 × 10^−3^ °C^−1^ for temperature sensing between 25 °C and 50 °C. The proposed sensor was tested on the human body to measure the temperature of various body parts, such as the forehead, neck, and palm. The sensor showed a consistent and reproducible temperature reading with a quick response and recovery time, exhibiting adequate capability to sense skin temperatures. This wearable sensor has the potential to be employed in a variety of applications, such as soft robotics, epidermal electronics, and soft human–machine interfaces.

## 1. Introduction

Recently, printed wearable biosensors have received significant attention in the healthcare and monitoring sectors [1,2]. Real-time data on health are obtained via the monitoring of vital biosignals, such as respiration rate, body temperature, and electrophysiology [3,4]. Ideally, these sensors are fabricated on biocompatible polymeric films and applied to the human skin. Particularly, these soft sensor patches are fabricated as e-tattoos and attached directly to the human skin or developed on highly deformable textile substrates [5,6,7]. Human body temperature is one of the key vital signs which has received special attention due to its capability of early indication of a variety of diseases [8,9]. Temperature sensors must be flexible, conformable, and attachable onto the human body for accurate measurement. Conventional temperature sensors are normally developed on rigid substrates and are not suited for soft wearable sensing applications. Conformable sensors cannot be realized using the conventional fabrication technologies involving high temperature and expensive vacuum-based processes, i.e., photolithography [10], e-beam lithography [11], chemical vapor deposition [12], atomic layer deposition [13], sputtering [14], etc. As a result, new printed electronics technologies have emerged which enable the direct fabrication of these electronic devices on various non-conventional soft substrates without the use of expensive conventional fabrication technologies [15,16,17,18]. For the fabrication of printed electronic devices, a variety of fabrication processes are available, including the sol–gel method [19], anodization [20], fused filament fabrication [21], roll-to-roll manufacturing [22], gravure printing [23], electrohydrodynamic printing [24,25], and piezo electric inkjet printing [26]. Among these printing technologies, inkjet printing is a promising non-contact approach for the efficient development of soft electronic devices at room temperature. Inkjet printing has the ability to produce high-resolution (~50 µm) structures with high repeatability, and film thickness in the range of tens of nanometers [9,25,27]. These capabilities make inkjet printing a useful fabrication tool to realize high-performance, soft wearable temperature sensors. 

Temperature sensors transform temperature changes into electrical signals with the use of sensing film and interdigitated electrodes (IDEs), or through a metallic meandering pattern. For temperature sensors, inkjet printing has so far enabled the realization of a variety of sensing materials and electrode designs. Simple soft sensors made of silver lines printed using an inkjet printer in a meandering pattern without any sensing film on polymeric films are demonstrated in [28,29]. A meandering micro-ribbon and an interdigitated electrode array were fabricated by inkjet printing silver nanoparticles ink and PEDOT:PSS onto photo glossy paper. The device monitored the temperature, relative humidity, and compressive and tensile bending all at once [30]. The developed sensor showed reasonable sensitivity for temperature over a wider range, but it was still crucial to optimize the geometric parameters of the sensor to further improve its performance. Furthermore, the developed device was not entirely wearable, so it was not readily useful for measuring the human body temperature. Similarly, adequate temperature sensitivity was demonstrated by a temperature sensor that was printed on flexible PET films using a mixture of carbon nanotube (CNT) ink and PEDOT:PSS solution. However, the demonstrated device was not entirely wearable [31]. In another study, a temperature sensor that was inkjet-printed on flexible plastic films using a mixture of carbon nanoparticle ink, dimethyl sulfoxide ink, and PEDOT:PSS solution was shown to have high linearity and low hysteresis for a wide range of temperatures. The presented device, however, was also not wearable, making it unsuitable for monitoring the body temperature of humans [32]. A self-powered and wearable printed temperature sensor has been developed utilizing PEDOT:PSS, silver nanoparticles, and graphene ink [33]. Despite having a decent sensing capability and a simple, inexpensive manufacturing technique, the sensor showed temperature-sensing characteristics that were reliant on the stretching orientations. Additionally, there is no geometric optimization of the sensing materials to enhance the performance. IDE-based sensors containing a sensing film of graphite and PEDOT:PSS composite are presented in [34,35,36]. Similar to this, a wearable temperature sensor with adequate sensitivity over a wide temperature range was printed on flexible and stretchable substrates using a mixture of polyhydroxybutyrate (PHB) as the matrix and reduced graphene oxide (rGO) as the nanofiller (PHB-rGO). This was accomplished by drop-coating biopolymer onto inkjet-printed silver electrodes [37]. The materials utilized to make the sensors demonstrated decent sensitivity, but to enhance their performance even more, the geometric parameters of the sensors need to be optimized. A doctor blade and inkjet printing technique were used to create a human body temperature sensor made of carbon black and silver IDEs on a flexible substrate [9]. The fabricated sensor exhibited reasonable sensitivity across a wide temperature range with quick response and recovery times, but there was no geometric optimization of the sensing materials to further improve the performance. Similarly, a human body soft temperature sensor made of CNTs has also been reported [38]. The fabricated sensor worked effectively when compared to conventional commercial platinum temperature sensors, but its performance is still limited and could be improved by optimizing the geometrical parameters of the sensors. Another study used a crosslinker (3-glycidyloxypropyl)trimethoxysilane (GOPS) and fluorinated polymer passivation (CYTOP) to significantly improve the temperature sensitivity of PEDOT:PSS film [39]. The developed sensor demonstrated reasonable sensitivity for temperature across a wider range, but it is still important to optimize the geometric parameters of the sensors to further improve their performance. The limitations of the above sensors, including their wearability, sensitivity to input temperature, and temperature measurement range, can be partially overcome by improving the materials and geometrical parameters of the sensors. Specifically, optimization of the electrode geometry and the size of the sensing film are significant concerns that need to be addressed for the widespread acceptance of these sensors in the wearable electronics industry. 

Here, we present a novel study on an IDE-based temperature sensor’s optimization for the temperature range of human skin. The sensor was fabricated on a polymeric substrate via the combination of low-cost inkjet and screen printing processes. The sensor consisted of silver IDE and carbon-black sensing film that are deposited on a 50 µm thick Kapton film substrate. The sensor was optimized for measuring the temperature of human skin by adjusting the finger spacing of the IDE from 0.1 to 1 mm and the width of the carbon film from 0.2 to 2 mm. After the optimization of the sensor’s geometric parameters (finger space 0.4 mm, film width 1.5 mm), the sensitivity (TCR) was enhanced to 3.93 × 10^−3^ °C^−1^ over a span of 25 to 50 °C, covering the entire temperature range of human skin. The optimized sensor also showed a fast response of 4.2 s and recovery time of 8.6 s. The proposed sensor was put to the test on the human body for continuous temperature monitoring of diverse body areas, including the forehead, palm, and neck. The sensor recorded stable and repeatable temperature readings, demonstrating its ability to measure human skin temperatures. We believe that this low-cost printed temperature sensor has potential to be used in numerous emerging applications such as soft robotics, epidermal electronics, and soft human–machine interfaces. 

## 2. Experimental Details 

### 2.1. Materials

IDEs were printed with silver nanoparticle ink obtained from Sigma-Aldrich and employed without any alterations. This silver nanoparticle ink was specially formulated for the DMP 2850 inkjet printer. Silver ink has nanoparticle loading of ~40% in ethylene glycol, resulting in high conductivity of the printed lines. Additionally, this ink has the optimal viscosity of ~10 cP needed for the inkjet printers. This ink can print as fine a line as 50 μm with high electrical conductivity. Typically, inks with viscosity in the range of 10 to 12 cP are suitable for this inkjet printer to generate micrometer size droplets with consistent volume. The designed .bmp files by ACE 3000 were then loaded into the DMP-2850 inkjet printing system. The printer converts the .bmp file into .ptn file format that is compatible with this printer. For the temperature-sensing film, we utilized carbon black powder, which was obtained from Sigma-Aldrich. The carbon black powder was mixed in ethylene glycol by a ratio of around 18 wt.%. The final ink formulation had a viscosity of around 22 cP, which was within the operating range of the screen printer. 

A Kapton film of around 50 µm was purchased from DuPont. This substrate was chosen due to its compatibility with our chosen inks, i.e., silver and carbon black, and due to its relatively high glass transition temperature. Other benefits of Kapton substrates for soft temperature sensors are their light weight, high temperature processing capability, and bendability. These features of Kapton make it an attractive substrate material for other soft wearable electronic devices and circuits. 

### 2.2. IDE Design 

**Figure 1:**
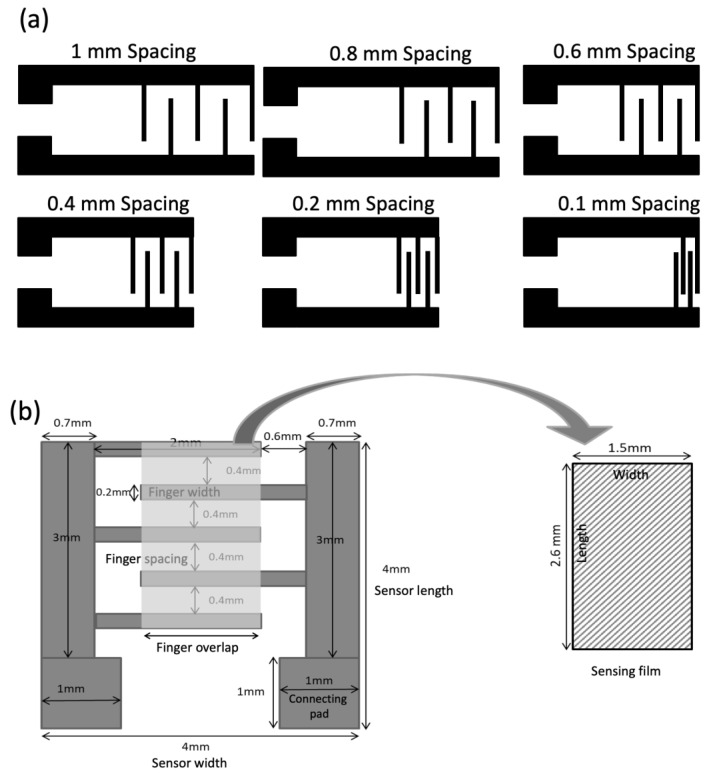
(**a**) Interdigitated electrode design with finger spacing from 0.1 to 1 mm, and (**b**) geometric parameters of the optimized sensor.

IDEs were digitally designed in a commercial CAD software (ACE 3000) which is compatible with our inkjet material printer DMP 2850. IDEs with various finger spacings were designed to choose the one among them which outperforms the others. The objective of this process was to obtain an optimum IDE design capable of having sensitivity to temperature. Finger spacings of the IDEs varied from 0.1 mm to 1 mm while the width of the finger was maintained constant at 200 µm to have high conductivity. Figure 1 displays the schematic diagram of the IDEs with finger spacing ranging from 0.1 mm to 1 mm. The designed IDEs were sent to the inkjet printer as a *.bmp* file for fabrication. All the fabricated IDEs were then coated with identical sensing films, and were tested on human skin. Then, the sensing performance of each IDE was analyzed and compared with others to achieve the optimum design. Based on this optimization, the optimal sensor IDE had a total size of 4 × 4 mm^2^, finger spacing of 0.4 mm, finger overlaps of 1.5 mm, and connecting pads of 1 × 1 mm^2^. 

### 2.3. Fabrication of the Sensor

**Figure 2:**
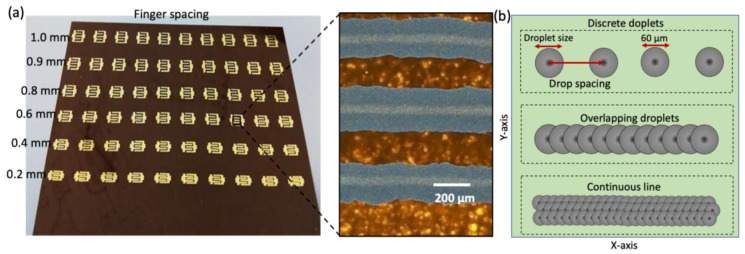
(**a**) The photograph and optical microscopic image of the fabricated silver IDEs on Kapton film, and (**b**) Schematic illustration of continuous line development caused by individual ink droplet overlap.

Kapton films were properly rinsed with acetone, isopropanol, and DI water, and underwent UV treatment for 5 min. This is an effective cleaning method to prepare the polymeric substrates for high-quality printing and to enhance the adhesion between the functional ink and the substrate. The IDEs were printed using a Dimatix (DMP 2850) inkjet printer, which has the ability to print at a high resolution (~50 µm) on a range of unconventional flexible substrates with a surface size of 210 × 315 mm^2^. Silver ink was filtered with a 5 µm filter and filled into the 3 mL cartridge reservoir. The cartridge has 16 nozzles and each one generates 10 pL droplets. The cartridge was then loaded into the printer head. A Kapton film was loaded on the printing stage and the vacuum of the platen was turned on. The targeted design file of the IDE was loaded together with the optimum printing parameters, i.e., stage temperature (30 °C), firing frequency (1000 Hz), and nozzles to substrate distance (100 µm). Printer settings were applied, such as drop velocity, which is the ink droplet’s velocity as it is ejected from the printing nozzle (6 mm/sec), along with stepping jet waveform and drop spacing (the distance between the centers of two subsequent drops) of 30 µm. These printing parameters were optimized based on the printing quality of the silver ink. IDEs were realized in two printing layers to enhance the pattern thickness, which improves their electrical conductivity and mechanical strength on the Kapton film, as shown in Figure 2a. For consistency, 10 IDEs for each finger spacing IDE were printed in a batch. Then, for the purpose of optimization, they were employed to fabricate sensors with various sensing film widths. The IDEs were randomly selected for the test because they exhibit comparable conductivities and were from the same batch. Figure 2b displays the droplet size, drop spacing, and drop overlap in a schematic illustration of the continuous line formation produced by each ink droplet overlap. The schematic in Figure 2b also illustrates that the drop spacing is along the printing’s x-axis. The printed IDEs were thermally sintered in an electric oven at 120 °C for 30 min, as advised by the ink manufacturer. After curing, the highly conductive IDEs were ready to use for further steps in the sensor fabrication.

**Figure 3:**
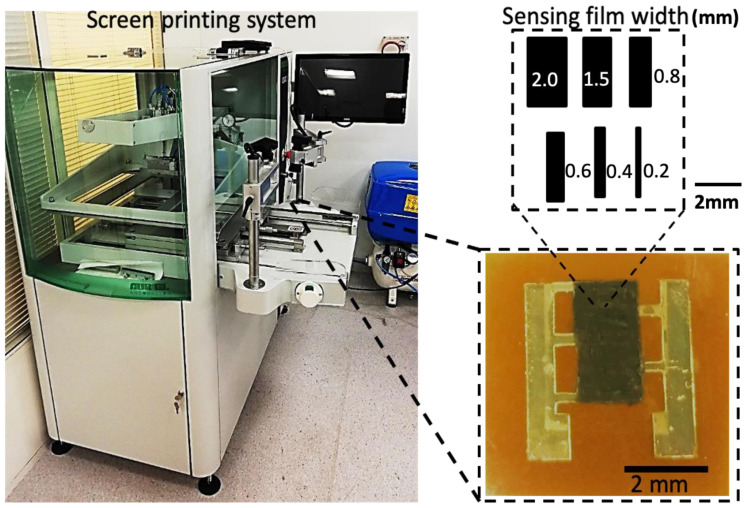
Photograph of the screen printer (Aurel Automation 2015A) used for deposition of the sensing films. The insets show the schematic diagram (top right) of the tested sensing films with various film widths (0.2 mm, 0.4 mm, 0.6 mm, 0.8 mm, 1.5 mm, and 2.0 mm) and a photograph (bottom right) of the final optimized device.

The IDEs were then loaded in the screen printer (Aurel Automation 2015A) platen for the deposition of the carbon-black-sensing film. A patterned mesh was placed on top of the IDEs with perfect alignment to fully cover the fingers’ overlapping area with carbon black. The screen printer parameters were optimized for a uniform film thickness. Figure 3 displays the screen printer used for the fabrication of the sensing film on top of the IDEs. After printing of the carbon film, the samples were thermally sintered at 120 °C in an electric oven for 30 min. Electrical connections for the resistance reading were established via a conducting epoxy (Chemtronics cw 2400) and copper wires. The schematic diagram of the sensing films and the photograph of the final fabricated sensor is presented in Figure 3. Encapsulation is an important step for ensuring a highly stable device since silver and the carbon films are highly prone to oxidation. The sensor was encapsulated via a two-part silicone epoxy. The device’s electrical resistance variation against temperature was stable after encapsulation. The finger spacing, sensing film width, and sensing film length are the three key factors that affect the performance of an IDE-based sensor for the same materials. The overall performance is least affected by the thickness of the silver IDE and carbon-sensing film. The thickness of the carbon layer was maintained throughout the experiments at around 10 μm. Thus, the sensor was optimized by adjusting the finger spacing (tested range of 0.1 mm to 1 mm, with 0.4 mm being the optimal spacing) and sensing film width (tested range of 0.2 to 2 mm, with 1.5 mm being the ideal width), while maintaining the same IDE and sensing film thicknesses. 

### 2.4. Characterizations of the Sensor

The surface morphology of the printed films was characterized via a scanning electronic microscope (JSM-7600F, Jeol, Tokyo, Japan), optical microscope (BX 41, Olympus, Tokyo, Japan), and 3D nano-profiler (contour-500, Bruker, Billerica, MA, USA). The electrical conductivity of the printed IDEs was determined through a four-point probe system (Ossila, T2001A3). For the conductivity measurement, four-point electrodes were placed on the sample covering a square area, and the resistance was recorded by using a source meter (B2900A, Keiysight, Santa Rosa, CA, USA). A variable temperature hot plate was used for heating up the sensor and the Keysight source meter was used for recording the resistance data.

## 3. Results and Discussion 

### 3.1. Structure of the Temperature Sensor 

**Figure 4:**
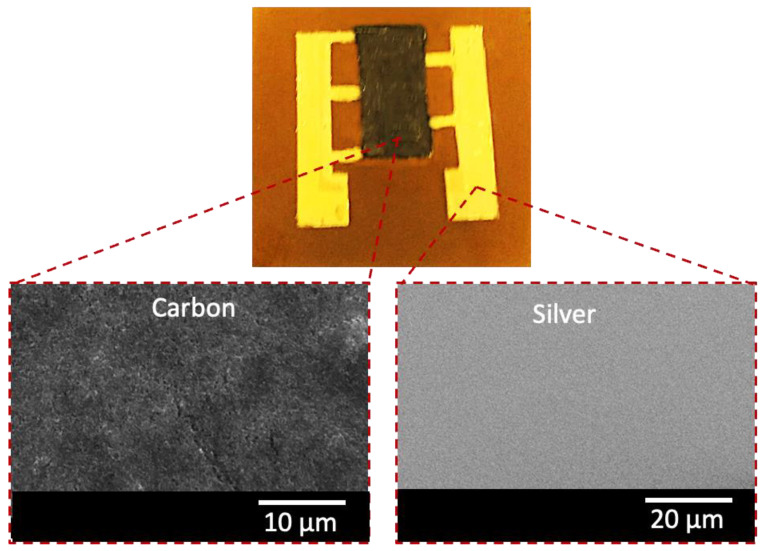
Photograph of the optimized temperature sensor (**top**) and the corresponding SEM images of the carbon film (**bottom left**) and silver IDEs (**bottom right**).

Surface morphology characterization is important to observe the physical features of the fabricated microstructures and to point out the imperfections. IDEs and sensing films were analyzed for surface morphology via SEM to confirm the film’s uniformity. Sample images of the silver IDE and carbon-sensing film are presented in Figure 4. These SEM images show that the IDE and sensing film both contain uniform microstructures, which contributed to the consistent performance of these films. 

### 3.2. IDE Geometric Optimization

**Figure 5:**
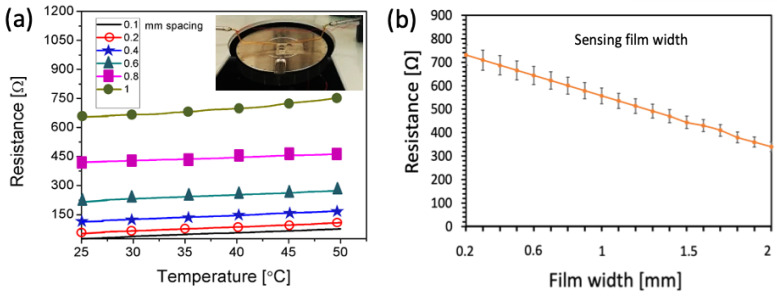
Electrical characterization of the devices. (**a**) Resistance variation with the temperature. (**b**) Resistance variation with sensing film width and an optimum finger spacing of 0.4 mm.

The effect of the IDE finger spacing was analyzed through resistance variation measurement of the sensor against temperature. For this analysis, all sensors, i.e., with 0.1, 0.2, 0.4, 0.6, 0.8, and 1 mm finger spacing and a carbon film thickness of 10 μm, were tested on a temperature scale from 25 to 50 °C. The sensors were heated using a variable temperature hot plate, and the source meter was used to record the data by electrically connecting to it using probes. For each type of sensor, the temperature varied from 25 to 50 °C, and the resistance data were recorded with 1000 data points. The inset of Figure 5a shows the resistance measurement setup where the sensor is mounted on a probe station and electrically coupled to the source meter to measure resistance in relation to each finger spacing. As is evident, practically all finger spacings exhibit a linear response in resistance against temperature; however, the base values are different from each other, as shown in Figure 5a. These reported resistance values are the averages of data collected from five individual sensors at each temperature level. Small finger spacings exhibited low base resistance, and base resistance increased as finger spacing increased. Above 0.8 mm spacing, the resistance variation was not linear, and this effect can be seen in the graph of the 1 mm spacing IDE. It was found that the appropriate spacing for temperature sensing applications is between 0.4 and 0.8 mm. However, the optimum finger spacing was found to be 0.4 mm in order to maintain the device size at 4 × 4 mm^2^. Based on sensor optimization for the smallest size, this device size was adopted. Smaller than these dimensions, the sensor’s contact area with the skin is insufficient to accurately measure its temperature. Additionally, it is difficult to manage a single device that is smaller than this size for the monitoring of human skin temperature. Figure 5b shows sensing film width optimization of the sensor (on top of an optimized IDE of 0.4 mm finger spacing), which, like the finger spacing effect, showed variation in the base resistance. As is evident, the base resistance is inversely proportional to the width of the sensing film. Any sensor film width between 0.2 and 2 mm demonstrated a linear response versus the temperature range. Due to the device’s size restriction, the optimum film was retained at 1.5 mm.

### 3.3. Testing for the Temperature 

RTDs (resistance temperature detectors) are contact-based temperature sensors that change in resistance as the temperature changes. The temperature of the measuring body is determined by the variation in resistance induced by temperature change. Small size, high accuracy, quick response time, and simple construction are major advantages of RTD-type temperature sensors. 

**Figure 6:**
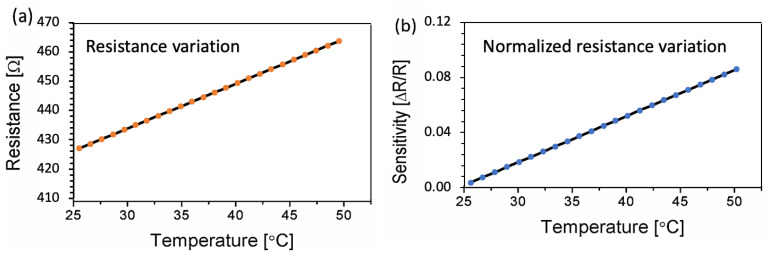
Electrical characterization of the temperature sensor. (**a**) Resistance variation of the sensor against temperature, ranging from 25 to 50 °C. (**b**) Corresponding sensitivity graph.

To determine the device’s durability against temperature fluctuations, the sensor was tested over a temperature range of 25 to 50 ° C. The sensor was deployed on a temperature-variable hot plate, and the sensor’s connections were made with the source meter. As illustrated in Figure 6a, the temperature of the hot plate was varied by increments of 1 °C, and the corresponding resistance was measured. The sensor’s behavior was found to be linear and repeatable over a temperature range of 25 to 50 °C. Carbon black responds linearly to temperature variation, acting as a resistor between the electrode fingers at lower temperature ranges such as 20–60 °C [40]. The electrical resistance of a carbon thin film inherently increases as the temperature increases. This is due to the increased thermal energy of the electrons in the film, which restricts them to move more freely and increases the resistance to electrical current flow [41,42]. Furthermore, our sensor is IDE-based, which improves the linearity of the resistance response to temperature since the carbon film between the two subsequent electrodes is rather small. Although the suggested sensor’s intended use is human skin temperature monitoring, it outperforms in the temperature range of 25 to 50 °C, expanding the sensor’s application to include robotic artificial skin. Equation (1) was used to calculate the sensor’s sensitivity from the observed resistance against temperature data. The sensor’s sensitivity data for the temperature range of 25 to 50 °C are shown in Figure 6b.

A parameter that is widely used to describe the sensitivity of temperature sensors is the temperature coefficient of resistance (TCR), which is described by the linear relationship between resistance and temperature. The TCR can be determined using Equation (1).

(1) $$TCR=\frac{R_{b}-R_{a}}{R_{a}\left(\Delta T\right)}$$

Here, $\Delta R=R_{b}-R_{a}$. *Ra* is the sensor’s initial resistance at 25 °C, *R_b_* is the current resistance at a specific temperature, ∆*T* =$\;T_{b}-T_{a}$ is the sensor’s temperature change, *T_a_* is the sensor’s initial temperature at 25 °C, and *T_b_* is the sensor’s current temperature. Using the same materials and design but without optimization, the value of TCR was reported to be 3.75 × 10^−3^ °C^−1^ [9]. The optimized sensor’s TCR is computed from the slope of Figure 6b using Equation (1), and the result shows adequate sensitivity to temperature changes with a calculated TCR of 3.93 × 10^−3^ °C^−1^. This optimization of IDEs’ finger spacing and the width of the carbon film improved the sensors’ sensitivity by 5% as compared to the initial design. These sensitivity and TCR data indicate that our optimized sensor is capable of making accurate temperature measurements over a broad temperature range between 25 °C and 50 °C.

Since diverse manufacturing techniques, materials, and sensor designs are used in temperature sensors, much like in other sensing devices, it is difficult to compare and analyze their performance. Table 1 compares our optimized printed temperature sensor to existing printed temperature sensors in terms of design, sensitivity, sensing layer type, substrate type, encapsulating material, and printing processes. It is evident that even though biocompatible carbon black is employed as the sensing film, which is inexpensive and simple to manufacture, our optimized sensors function well in comparison, exhibiting a decent TCR value. This is partially due to the carbon black film’s optimal geometry and the silver IDE’s optimized design. The data in Table 1 clearly show that an exact comparison of the sensitivity and TCR of all temperature sensors mentioned in the literature is difficult. It is not entirely known where the performance variations originate since nearly all sensors employ different designs, sensing materials, substrates, and printing techniques; consequently, further research is needed to provide a comprehensive analysis.

### 3.4. Testing on Human Body 

**Figure 7:**
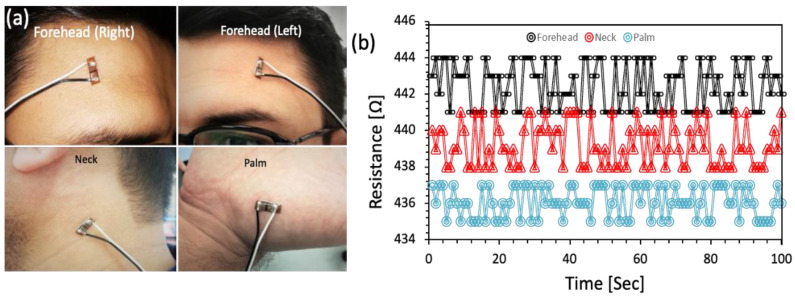
Sensor endurance test on human skin: (**a**) photographs of the sensors on different parts of the body (foreheads, neck, palm); (**b**) the corresponding resistance variations against temperature.

The temperature parameters of the sensor were examined to determine the real-time temperature of human skin. For this test, the temperature sensor was attached to the skin using double-sided thin tape. The sensor was placed on various spots to achieve the best response to the human skin. The sensor was placed on both sides of the forehead, on the neck, and on the palm, as shown in Figure 7a. Figure 7b displays the temperature resistance against temperature on the palm, forehead, and neck, among other body areas. The various colors depict various bodily components and their resistance on the Y-axis. The sensor measured a resistance of ~436 Ω at the human palm, ~440 Ω at the neck, and ~443 Ω at the forehead. The sensor was put through 10 endurance cycles, each lasting 20 min, to see how stable it was against human body temperature. The uncertainty in the resistance was measured to be ±1 Ω during the test, as shown in Figure 7b. 

### 3.5. Recovery and Response Time

**Figure 8:**
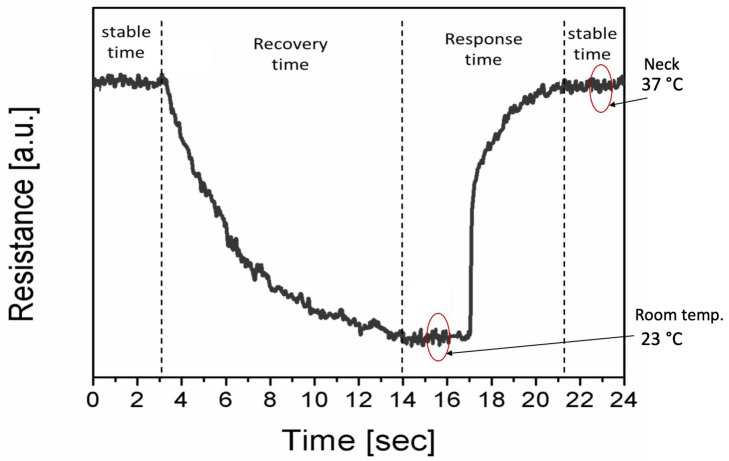
The cyclic response and recovery time of the temperature sensor.

The sensor’s response and recovery time were also investigated. A sensor was coupled to a source meter and put on a human neck to monitor the response time. The resistance of the sensors was initially modest, but as the temperature increased, it rose and became steady. Figure 8 displays the 4 s reaction time that was attained. It is the time required for the low-to-high resistance change (on the time scale from 17 to 21 s). The sensor’s resistance stabilized after 4 s, indicating the temperature value. To determine the recovery period, the sensor was detached from the neck and the resistance transition from high to low resistance was timed. The recovery time was estimated to be nearly 8.5 s, as shown in Figure 8. The sensor’s fast response and recovery time are promising for the human body temperature measurement as well as for the robotic skin.

## 4. Conclusions

We present a novel study on the optimization of a low-cost, printed, wearable human skin temperature sensor that was entirely screen-printed and low-cost inkjet-printed on a flexible plastic substrate. Inkjet printing of silver IDEs and screen printing of a carbon-black-sensing film were both optimized to achieve high sensitivity. The carbon film widths were adjusted from 0.2 to 2 mm and the finger spacing of the IDEs was changed from 0.1 to 1 mm to optimize the sensor geometry for the measurement of human skin temperature. The optimized sensor had an improved sensitivity of 3.93 × 10^−3^ °C^−1^ over a wide temperature range of 25 to 50 °C, which fully covered the range of temperatures of human skin. It also had a quick response time of 4.2 s and a recovery time of 8.6 s. The proposed sensor was then tested on the human body for real-time temperature measurement of different areas, such as the forehead, palm, and neck, where it recorded steady and consistent temperature readings, proving its ability to monitor the temperature of human skin. We believe this low-cost, inkjet-printed temperature sensor has considerable potential for applications in a wide range of cutting-edge domains, including but not limited to soft robotics, epidermal electronics, and soft human–machine interfaces.

## Figures and Tables

**Table 1:** Comparison of the performance of various flexible printed temperature sensors.

S. No.	Sensor Type	TCR/Sensitivity	Substrate	Printing Method	Sensing Film	Encapsulation	Ref.
1	RTD	0.6 × 10^−3^ °C^−1^	Cellulose	E-jet Printing	Silver	NA	[43]
2	RTD	2.8 × 10^−3^ °C^−1^	Polyurethane	Transfer Printing	Gold/Chromium	Polyurethane	[1]
3	RTD	2.2 × 10^−3^ °C^−1^	Polymide	Inkjet Printing	Silver	NA	[29]
4	RTD	11.1 × 10^−3^ °C^−1^	PET	E-jet Printing	Silver	NA	[44]
5	RTD	0.83 × 10^−3^ °C^−1^	Paper	Inkjet Printing	Silver	NA	[30]
6	Thermistor	2.5 × 10^−3^ °C^−1^	PET	Screen Printing	PEDOT:PSS	PDMS	[45]
7	Thermistor	8.9 × 10^−3^ °C^−1^	PET	Inkjet Printing	PEDOT:PSS/CNTs	Polymer resist	[31]
8	Thermistor	2.5 × 10^−3^ °C^−1^	PEN	Inkjet Printing	PEDOT:PSS/Carbon	NA	[32]
9	Thermistor	3.0 × 10^−3^ °C^−1^	PDMS	Inkjet Printing	PHB-rGO	PDMS	[37]
10	Thermistor	7.7 × 10^−3^ °C^−1^	PEN	Inkjet Printing	Silver/PEDOT:PSS	CYTOP	[39]
11	Thermistor	6.3 × 10^−3^ °C^−1^	PET	Screen Printing	PEDOT:PSS/CNTs	NA	[46]
12	Thermistor	10.9 × 10^−3^ °C^−1^	PVC	Shadow Masking	PEDOT:PSS/GO	NA	[47]
13	Thermistor	3.75 × 10^−3^ °C^−1^	PI	Inkjet Printing	Carbon	Silicone	[9]
14	Thermistor	3.93 × 10^−3^ °C^−1^	Kapton	Inkjet Printing, Screen Printing	Carbon	NA	This Work

## Data Availability

No data are available.

## References

[1] Chen Y., Lu B., Chen Y., Feng X. (2015). Breathable and Stretchable Temperature Sensors Inspired by Skin. Sci. Rep..

[2] Kim J., Campbell A.S., de Ávila B.E.-F., Wang J. (2019). Wearable biosensors for healthcare monitoring. Nat. Biotechnol..

[3] Ali S., Khan S., Khan A., Bermak A. (2020). Developing conductive fabric threads for human respiratory rate monitoring. IEEE Sens. J..

[4] Kenry, Yeo J.C., Lim C.T. (2016). Emerging flexible and wearable physical sensing platforms for healthcare and biomedical applications. Microsyst. Nanoeng..

[5] Cesarelli G., Donisi L., Coccia A., Amitrano F., D’Addio G., Ricciardi C. (2021). The E-Textile for Biomedical Applications: A Systematic Review of Literature. Diagnostics.

[6] Yang J.C., Mun J., Kwon S.Y., Park S., Bao Z., Park S. (2019). Electronic Skin: Recent Progress and Future Prospects for Skin-Attachable Devices for Health Monitoring, Robotics, and Prosthetics. Adv. Mater..

[7] Khan A., Roo J.S., Kraus T., Steimle J. Soft inkjet circuits: Rapid multi-material fabrication of soft circuits using a commodity inkjet printer. Proceedings of the 32nd Annual ACM Symposium on User Interface Software and Technology.

[8] Yao S., Swetha P., Zhu Y. (2018). Nanomaterial-Enabled Wearable Sensors for Healthcare. Adv. Healthc. Mater..

[9] Ali S., Khan S., Bermak A. (2019). Inkjet-Printed Human Body Temperature Sensor for Wearable Electronics. IEEE Access.

[10] Stevenson J.T.M., Gundlach A.M. (1986). The application of photolithography to the fabrication of microcircuits. J. Phys. E Sci. Instrum..

[11] Chen Y. (2015). Nanofabrication by electron beam lithography and its applications: A review. Microelectron. Eng..

[12] Sun L., Yuan G., Gao L., Yang J., Chhowalla M., Gharahcheshmeh M.H., Gleason K.K., Choi Y.S., Hong B.H., Liu Z. (2021). Chemical vapour deposition. Nat. Rev. Methods Prim..

[13] George S.M. (2010). Atomic Layer Deposition: An Overview. Chem. Rev..

[14] Kelly P.J., Arnell R.D. (2000). Magnetron sputtering: A review of recent developments and applications. Vacuum.

[15] Khan S., Ali S., Khan A., Bermak A. (2022). Developing pressure sensors from impregnated textile sandwiched in inkjet-printed electrodes. J. Mater. Sci. Mater. Electron..

[16] Wiklund J., Karakoç A., Palko T., Yiğitler H., Ruttik K., Jäntti R., Paltakari J. (2021). A Review on Printed Electronics: Fabrication Methods, Inks, Substrates, Applications and Environmental Impacts. J. Manuf. Mater. Process..

[17] Khan S., Ali S., Khan A., Ahmed M., Wang B., Bermak A. (2021). Inkjet printing of multi-stripes based deflection monitoring sensor on flexible substrate. Sens. Actuators A Phys..

[18] Leenen M.A.M., Arning V., Thiem H., Steiger J., Anselmann R. (2009). Printable electronics: Flexibility for the future. Phys. Status Solidi (A).

[19] Gergel-Hackett N., Hamadani B., Dunlap B., Suehle J., Richter C., Hacker C., Gundlach D. (2009). A flexible solution-processed memristor. IEEE Electron Device Lett..

[20] Miller K., Nalwa K.S., Bergerud A., Neihart N.M., Chaudhary S. (2010). Memristive behavior in thin anodic titania. IEEE Electron Device Lett..

[21] Kalaš D., Šíma K., Kadlec P., Polanský R., Soukup R., Řeboun J., Hamáček A. (2021). FFF 3D Printing in Electronic Applications: Dielectric and Thermal Properties of Selected Polymers. Polymers.

[22] Sirringhaus H., Kawase T., Friend R., Shimoda T., Inbasekaran M., Wu W., Woo E.P. (2000). High-resolution inkjet printing of all-polymer transistor circuits. Science.

[23] Robinson J.T., Burgess J.S., Junkermeier C.E., Badescu S.C., Reinecke T.L., Perkins F.K., Zalalutdniov M.K., Baldwin J.W., Culbertson J.C., Sheehan P.E. (2010). Properties of fluorinated graphene films. Nano Lett..

[24] Khan A., Rahman K., Kim D.S., Choi K.H. (2012). Direct printing of copper conductive micro-tracks by multi-nozzle electrohydrodynamic inkjet printing process. J. Mater. Process. Technol..

[25] Khan A., Rahman K., Ali S., Khan S., Wang B., Bermak A. (2021). Fabrication of circuits by multi-nozzle electrohydrodynamic inkjet printing for soft wearable electronics. J. Mater. Res..

[26] Seifert T., Sowade E., Roscher F., Wiemer M., Gessner T., Baumann R.R. (2015). Additive manufacturing technologies compared: Morphology of deposits of silver ink using inkjet and aerosol jet printing. Ind. Eng. Chem. Res..

[27] Zikulnig J., Hirschl C., Rauter L., Krivec M., Lammer H., Riemelmoser F., Roshanghias A. (2019). Inkjet printing and characterisation of a resistive temperature sensor on paper substrate. Flex. Print. Electron..

[28] Ali S., Hassan A., Bae J., Lee C.H., Kim J. (2016). All-printed differential temperature sensor for the compensation of bending effects. Langmuir.

[29] Dankoco M., Tesfay G., Bènevent E., Bendahan M. (2016). Temperature sensor realized by inkjet printing process on flexible substrate. Mater. Sci. Eng. B.

[30] Barmpakos D., Tsamis C., Kaltsas G. (2020). Multi-parameter paper sensor fabricated by inkjet-printed silver nanoparticle ink and PEDOT:PSS. Microelectron. Eng..

[31] Yamamoto Y., Harada S., Yamamoto D., Honda W., Arie T., Akita S., Takei K. (2016). Printed multifunctional flexible device with an integrated motion sensor for health care monitoring. Sci. Adv..

[32] Bali C., Brandlmaier A., Ganster A., Raab O., Zapf J., Hübler A. (2016). Fully Inkjet-Printed Flexible Temperature Sensors Based on Carbon and PEDOT:PSS1. Mater. Today Proc..

[33] Jung M., Jeon S., Bae J. (2018). Scalable and facile synthesis of stretchable thermoelectric fabric for wearable self-powered temperature sensors. RSC Adv..

[34] Shih W.-P., Tsao L.-C., Lee C.-W., Cheng M.-Y., Chang C., Yang Y.-J., Fan K.-C. (2010). Flexible temperature sensor array based on a graphite-polydimethylsiloxane composite. Sensors.

[35] Yin X., Wang W., Yu Y., Geng Y., Li X. (2015). Temperature Sensor Based on Quantum Dots Solution Encapsulated in Photonic Crystal Fiber. IEEE Sens. J..

[36] Liu G., Tan Q., Kou H., Zhang L., Wang J., Lv W., Dong H., Xiong J. (2018). A flexible temperature sensor based on reduced graphene oxide for robot skin used in internet of things. Sensors.

[37] Dan L., Elias A.L. (2020). Flexible and Stretchable Temperature Sensors Fabricated Using Solution-Processable Conductive Polymer Composites. Adv. Healthc. Mater..

[38] Kuzubasoglu B.A., Sayar E., Cochrane C., Koncar V., Bahadir S.K. (2021). Wearable temperature sensor for human body temperature detection. J. Mater. Sci. Mater. Electron..

[39] Wang Y.-F., Sekine T., Takeda Y., Yokosawa K., Matsui H., Kumaki D., Shiba T., Nishikawa T., Tokito S. (2020). Fully Printed PEDOT:PSS-based Temperature Sensor with High Humidity Stability for Wireless Healthcare Monitoring. Sci. Rep..

[40] Sarma S., Lee J.H. (2018). Developing Efficient Thin Film Temperature Sensors Utilizing Layered Carbon Nanotube Films. Sensors.

[41] Zhu G., Wang F., Chen L., Wang C., Xu Y., Chen J., Chang X., Zhu Y. (2022). Highly flexible TPU/SWCNTs composite-based temperature sensors with linear negative temperature coefficient effect and photo-thermal effect. Compos. Sci. Technol..

[42] Li C., Yang S., Guo Y., Huang H., Chen H., Zuo X., Fan Z., Liang H., Pan L. (2021). Flexible, multi-functional sensor based on all-carbon sensing medium with low coupling for ultrahigh-performance strain, temperature and humidity sensing. Chem. Eng. J..

[43] Vuorinen T., Laurila M.-M., Mangayil R., Karp M., Mäntysalo M. (2018). High Resolution E-Jet Printed Temperature Sensor on Artificial Skin. Proceedings of the EMBEC & NBC 2017, European Medical and Biological Engineering Confernce.

[44] Ahmad S., Rahman K., Cheema T.A., Shakeel M., Khan A., Bermak A. (2022). Fabrication of Low-Cost Resistance Temperature Detectors and Micro-Heaters by Electrohydrodynamic Printing. Micromachines.

[45] Harada S., Kanao K., Yamamoto Y., Arie T., Akita S., Takei K. (2014). Fully Printed Flexible Fingerprint-like Three-Axis Tactile and Slip Force and Temperature Sensors for Artificial Skin. ACS Nano.

[46] Harada S., Honda W., Arie T., Akita S., Takei K. (2014). Fully Printed, Highly Sensitive Multifunctional Artificial Electronic Whisker Arrays Integrated with Strain and Temperature Sensors. ACS Nano.

[47] Soni M., Bhattacharjee M., Ntagios M., Dahiya R. (2020). Printed Temperature Sensor Based on PEDOT: PSS-Graphene Oxide Composite. IEEE Sens. J..

